# Optimizing Long-Term Outcomes in Cystinosis With Comprehensive Patient-Centered Care

**DOI:** 10.1016/j.ekir.2024.10.038

**Published:** 2025-03-04

**Authors:** Ewa Elenberg

**Affiliations:** 1 Texas Children’s Hospital and Baylor College of Medicine, Houston, Texas, USA

## Introduction

Cystinosis is a rare autosomal recessive lysosomal storage disorder with an estimated incidence of 1 in 100,000 to 200,000 live births, though this is likely underestimated.^1^^,^^2^ It is caused by pathogenic variants in the *CTNS* gene on chromosome 17 that encodes for the transporter protein cystinosin resulting in defective transit of cystine out of lysosomes, cellular cystine accumulation, and subsequent multiorgan damage.^1^^,^^3^ Subtypes of cystinosis—nephropathic (infantile and intermediate/juvenile) and nonnephropathic (ocular)—have been described and vary based on age at presentation, organ involvement, and symptom progression. Infantile nephropathic cystinosis is the most common and severe form of the disease, with patients typically presenting with Fanconi syndrome, growth failure, and hypophosphatemic rickets in the first 6 to 12 months of life. Without treatment, patients with infantile nephropathic cystinosis progress to kidney failure by the age of 10 years and develop photophobia and blepharospasm from corneal cystine deposits.^1^^,^^2^ Intermediate/juvenile nephropathic cystinosis is diagnosed later in childhood or adolescence and has a milder phenotype than the infantile form, whereas the nonnephropathic subtype only affects the eyes.^1^, ^2^, ^3^ Once suspected, diagnosis of cystinosis is confirmed by white blood cell cystine level measurement, genetic testing for *CTNS* variants, and/or slit-lamp eye examination.^1^^,^^2^^,^^4^ Henceforth, we will use the term “cystinosis” to mean “infantile nephropathic cystinosis,” because such generalization is common practice in the literature due to the rarity of other forms.

**Figure 1:**
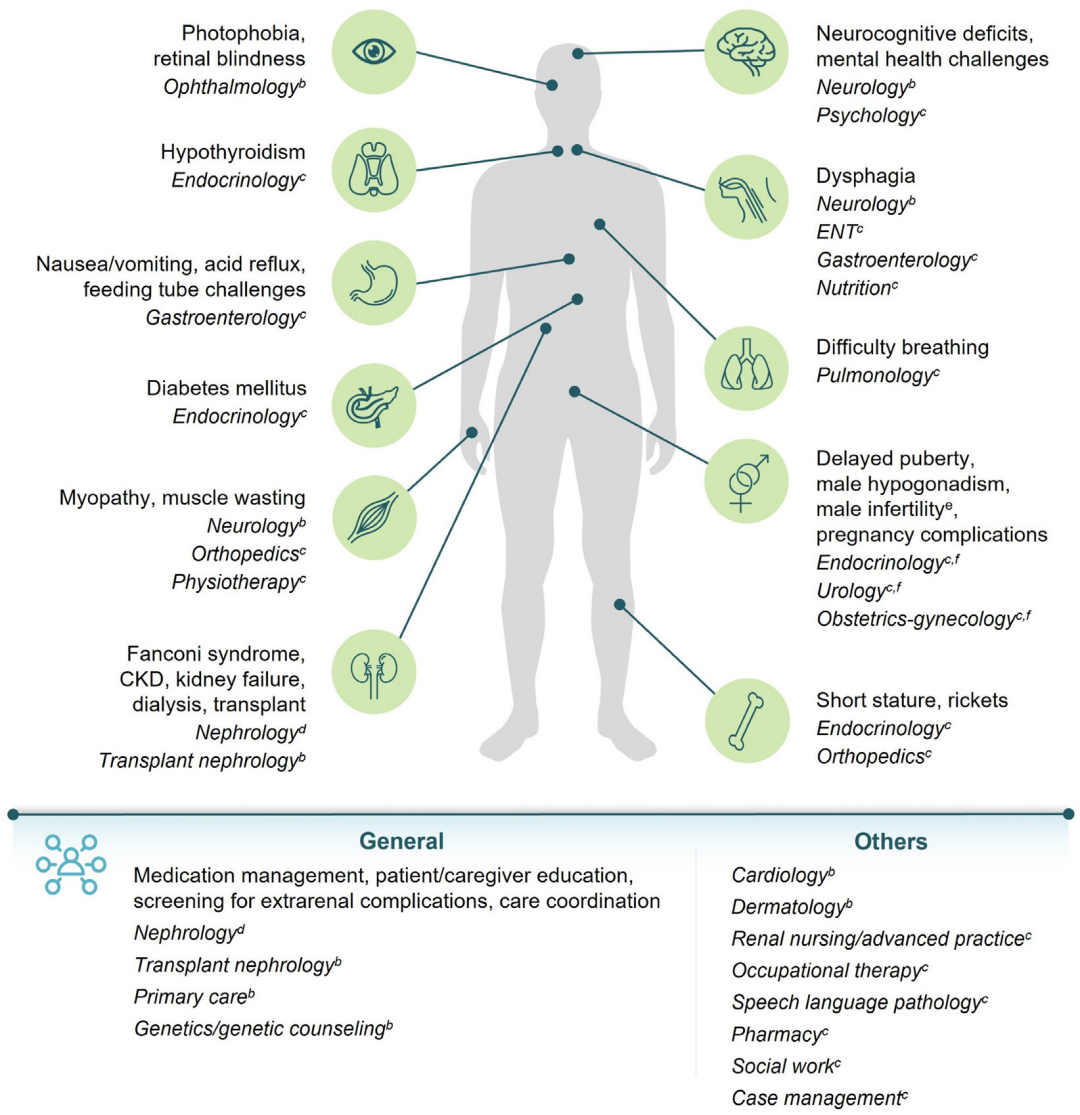
The multisystemic nature of cystinosis requires a multidisciplinary approach to effectively monitor and address complications in adulthood. General or transplant nephrology teams typically act as “care quarterbacks” to coordinate complex management across specialties. Common disease manifestations, particularly in untreated and undertreated patients, are listed in Figure 1, along with specialties which may manage these conditions and suggested follow-up timing.^1^^,^^2^^,^^4^, ^5^, ^6^, ^7^^,^S5^,^S6^,^^a^ ^a^Multispecialty involvement and follow-up frequency should be tailored to individual patient needs.^4^^,^^6^^,^S5^,^S6 ^b^Follow-up at least once annually.^6^^,^S5^,^S6 ^c^Follow-up as appropriate.^6^^,^S5^,^S6 ^d^Follow-up at least twice annually.S5 ^e^Cystinosis has not been shown to cause infertility in women.^2^^,^^4^^,^^6^^,^S5 ^f^Including fertility specialists.S5 CKD, chronic kidney disease; ENT, otolaryngology.

Earlier diagnosis, advances in transplantation, and targeted treatment with cysteamine, which depletes lysosomal cystine, have transformed cystinosis from a fatal pediatric kidney disorder to a chronic multisystemic condition, with patients living longer into adulthood.^1^, ^2^, ^3^ Although the disease does not recur in the allograft, transplantation does not correct the underlying metabolic defect in cystinosis.^1^^,^^2^ Cystine continues to accumulate in extrarenal tissues, and by adulthood, nearly every organ system may be impacted. Particularly in the absence of adequate treatment, sequelae such as hypothyroidism, diabetes, male hypogonadism and infertility, metabolic bone disease, gastrointestinal dysmotility, hepatosplenomegaly, myopathy, neurocognitive impairment, keratopathy, and retinopathy, among others, can manifest over time (Figure 1).^1^^,^^2^^,^^5^, ^6^, ^7^

**Figure 2:**
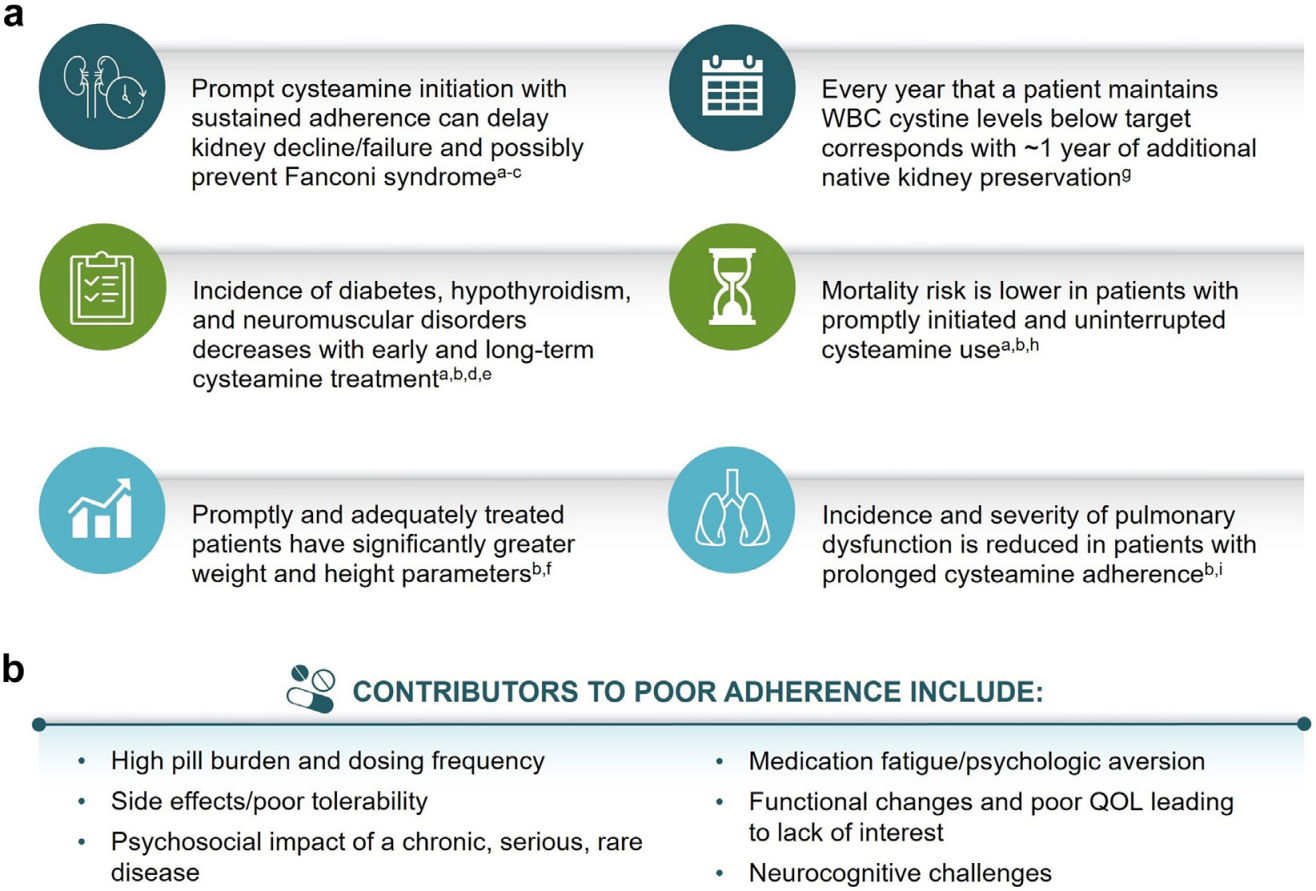
Early and continuous cysteamine treatment is essential to prevent or delay complications in patients with cystinosis. (a) Select patient outcomes with early and long-term oral cysteamine therapy.^3^^,^^7^, ^8^, ^9^^,^S1^,^S2 (b) Select contributing factors to medication nonadherence in patients with cystinosis.^2^^,^^4^^,^S5 ^a^Early/prompt cysteamine initiation was defined as before 5 years of age.^9^ ^b^Long-term/adequate treatment was defined as receiving cysteamine for ≥8 years.^3^ ^c^Fanconi syndrome was attenuated with cysteamine initiation within 2 months of birth after identification of cystinosis owing to an affected sibling or newborn screening.^8^ ^d^Neuromuscular disorders included myopathy, dysphagia, paresis, cerebrovascular accident, mental function deterioration, and seizures.^9^ ^e^Starting therapy after the age of 5 years still delayed hypothyroidism and diabetes when compared with untreated patients.^9^ ^f^Prompt cysteamine treatment was defined as before 1.5 years of age.S2 ^g^The target granulocyte level is < 1.9 nmol ½ cystine/mg protein, and the target mixed leukocyte level is < 1.0 nmol ½ cystine/mg protein. Tests are not interchangeable.S1^,^S7 ^h^Starting therapy after 5 years of age still improved life expectancy when compared with untreated patients.^9^ ^i^Pulmonary insufficiency, swallowing dysfunction, and aspiration cause significant morbidity and mortality in adults with cystinosis.^3^^,^^9^ QOL, quality of life; WBC, white blood cell.

Comprehensive management of cystinosis consists of supportive therapies for Fanconi syndrome, chronic kidney disease or kidney failure, and extrarenal complications, as well as specific treatment with cysteamine.^1^^,^^2^ Continuous, long-term cysteamine therapy initiated promptly after diagnosis has been shown to improve life expectancy, preserve growth, delay kidney decline, and limit extrarenal manifestations associated with this disorder (Figure 2).^3^^,^^7^, ^8^, ^9^^,^S1^,^S2 Cysteamine eye drops are also necessary because oral formulations are ineffective at reducing corneal cystine accumulation.^1^^,^^2^ In addition to aiding in cystinosis diagnosis, white blood cell cystine level testing is used as a surrogate to inform cystine control, cysteamine adherence, and the need for dosage adjustments. However, these levels only represent a snapshot of cystine depletion, may not adequately reflect tissue cystine concentrations, and are heavily dependent on when the last cysteamine dose was taken, making it difficult to accurately assess long-term disease control based on this testing alone.^4^ Alternative biomarkers, such as chitotriosidase, are being evaluated for potential utility in therapeutic monitoring in cystinosis.S3^,^S4

Given the complexity of cystinosis and its multisystemic impacts, in this *Kidney International Reports* supplement, we aim to illustrate the importance of supporting early and continuous cystine control with cysteamine treatment, coordinating management with multidisciplinary clinicians, facilitating transitions of care, and ultimately improving patient outcomes.

## Case Presentations

The case presented by Benfield describes 2 siblings—Patients A and B—who were diagnosed with cystinosis and initiated cysteamine therapy at the ages of 3 and 1.5 years, respectively. Patient A, who is currently 13 years old, experienced severe Fanconi syndrome with significant fluid and electrolyte imbalances requiring repeat hospitalizations. Patient B, who is currently 11 years old, has been relatively stable with minimal medical intervention. The divergent patient outcomes in this case demonstrate the urgency of early diagnosis and prompt treatment initiation in limiting long-term deterioration in cystinosis. The author also discusses the unmet need for improved testing via newborn screening and in siblings with a known family history of cystinosis, as well as the impact of rare diseases on caregivers.

The case outlined by Ghossein and Nishi explores a successful approach for facilitating the transition to adult cystinosis care. A 23-year-old patient was transferred from pediatric to adult nephrology providers via a specialized transition clinic and received ongoing multidisciplinary support to monitor and address extrarenal complications, including myopathic and orthopedic challenges, hypothyroidism, worsening executive functioning, and visual impairment. The patient’s white blood cell cystine levels were above target around the time of transition, though the patient denied extended periods of nonadherence, and the adult nephrology team continues to monitor levels and titrate the oral cysteamine dosage accordingly. This case highlights the importance of developing patient disease- and self-management skills before transition, clinician recognition of the effects of transfer timing on medication adherence and care engagement, as well as continuity of collaborative and coordinated care with multidisciplinary specialists.

Jarnes *et al.* further underscore the variability of extrarenal manifestations in cystinosis and the need for individualized and comprehensive management. The case presents a 36-year-old patient currently managed at a metabolic genetics clinic who was diagnosed with cystinosis later than average at 5 years of age, with cysteamine therapy initiated shortly thereafter. Though white blood cell cystine levels indicate disease control, it has been difficult for the patient to fully tolerate prescribed oral cysteamine dosages due to gastrointestinal symptoms. The patient also experiences progressive complications that limit quality of life and ability to work, including pain, fatigue, and migraine. The authors discuss the implications of delayed diagnosis on long-term outcomes and the impact of patient advocacy and involvement in the cystinosis community on psychosocial well-being.

## Conclusion

In summary, the 3 original case reports presented in this supplement collectively highlight the importance of early diagnosis, prompt treatment initiation, and lifelong comprehensive care to support the evolving needs of patients with cystinosis. Additional themes include continued medication adherence and care engagement, ongoing disease education, development of self-management skills, multispecialty management of extrarenal manifestations, the impact of treatment decisions on quality of life, and the role of patient advocacy. As patients with cystinosis live longer into adulthood, more research is needed to sufficiently address challenges across the life span and reduce morbidity and mortality.

## Disclosure

EE has received honoraria from Amgen Inc (formerly Horizon Therapeutics plc) for past consulting/advisory activities.

## Patient Consent

The author declares that consent was obtained from the patients and/or families of the patients discussed in the reports.

## Supplementary Material

Supplementary File (PDF) **Contributing Authors. Supplementary References**.

## Footnotes

Supplementary File (PDF)

**Contributing Authors.**

**Supplementary References.**

## References

[1] Gahl W.A., Thoene J.G., Schneider J.A. (2002). Cystinosis. N Engl J Med.

[2] Nesterova G., Gahl W. (2008). Nephropathic cystinosis: late complications of a multisystemic disease. Pediatr Nephrol.

[3] Gahl W.A., Balog J.Z., Kleta R. (2007). Nephropathic cystinosis in adults: natural history and effects of oral cysteamine therapy. Ann Intern Med.

[4] Langman C.B., Barshop B.A., Deschênes G. (2016). Controversies and research agenda in nephropathic cystinosis: conclusions from a “Kidney Disease: improving Global Outcomes” (KDIGO) Controversies Conference. Kidney Int.

[5] Kasimer R.N., Langman C.B. (2021). Adult complications of nephropathic cystinosis: a systematic review. Pediatr Nephrol.

[6] Topaloglu R. (2024). Extrarenal complications of cystinosis. Pediatr Nephrol.

[7] Ariceta G., Giordano V., Santos F. (2019). Effects of long-term cysteamine treatment in patients with cystinosis. Pediatr Nephrol.

[8] Hohenfellner K., Nießl C., Haffner D. (2022). Beneficial effects of starting oral cysteamine treatment in the first 2 months of life on glomerular and tubular kidney function in infantile nephropathic cystinosis. Mol Genet Metab.

[9] Brodin-Sartorius A., Tête M.-J., Niaudet P. (2012). Cysteamine therapy delays the progression of nephropathic cystinosis in late adolescents and adults. Kidney Int.

