# Supplementary material for: Optimizing Long-Term Outcomes in Cystinosis With Comprehensive Patient-Centered Care
Source: Kidney Int Rep. 2025 Mar 4;10(3 Suppl):S775–8. doi: 10.1016/j.ekir.2024.10.038 (PMC11935153; doi:10.1016/j.ekir.2024.10.038)
Supplement: Supplementary File (PDF) — Contributing Authors. Supplementary References. [file mmc1.pdf]

CONTRIBUTING AUTHORS

SUPPLEMENT: Optimizing long-term outcomes in cystinosis with comprehensive patient-centered care

TABLE OF CONTENTS

Article type	Title	Authors	Affiliations
Editorial/ overview	Optimizing long-term outcomes in cystinosis with comprehensive patient-centered care	Ewa Elenberg, MD, MEd	Texas Children's Hospital and Baylor College of Medicine *Houston, TX*
Nephrology rounds/ case report	Impact of early versus late diagnosis on disease progression in cystinosis	Mark R. Benfield, MD	Pediatric Nephrology of Alabama, PC *Birmingham, AL*
Nephrology rounds/ case report	Transitioning care in nephropathic cystinosis: overcoming challenges in young adults	Cybele Ghossein, MD<br>Laura Nishi, DScPAS, PA-C	Feinberg School of Medicine, Northwestern University *Chicago, IL*
Nephrology rounds/ case report	Addressing the multisystemic impacts of nephropathic cystinosis in an adult	Jeanine R. Jarnes, PharmD, BCOP, BCPS<br>Chester B. Whitley, PhD, MD<br>Rebekah S. Palmer, BS	University of Minnesota, Department of Pediatrics *Minneapolis, MN*<br>Next Generation of Cystinosis *Ankeny, IA*

SUPPLEMENTARY REFERENCES

S1. Nesterova G, Williams C, Bernardini I, et al. Cystinosis: renal glomerular and renal tubular function in relation to compliance with cystine-depleting therapy. *Pediatr Nephrol*. 2015;30(6):945-951. https://doi.org/10.1007/s00467-014-3018-x

S2. Emma F, Hoff VW, Hohenfellner K, et al. An international cohort study spanning five decades assessed outcomes of nephropathic cystinosis. *Kidney Int*. 2021;100(5):1112-1123. https://doi.org/10.1016/j.kint.2021.06.019

S3. Emma F, Montini G, Pennesi M, et al. Biomarkers in nephropathic cystinosis: current and future perspectives. *Cells*. 2022;11(11):1839. https://doi.org/10.3390/cells11111839

S4. Veys K, Elmonem MA, van den Heuvel L, et al. Plasma chitotriosidase enzyme activity as a novel therapeutic monitor for cysteamine treatment in nephropathic cystinosis: a retrospective validation study. *Mol Genet Metab*. 2024;142(1):108454. https://doi.org/10.1016/j.ymgme.2024.108454

S5. Levtchenko E, Servais A, Hulton SA, et al. Expert guidance on the multidisciplinary management of cystinosis in adolescent and adult patients. *Clin Kidney J*. 2022;15(9):1675-1684. https://doi.org/10.1093/ckj/sfac099

S6. Raina R, Wang J, Krishnappa V. Structured transition protocol for children with cystinosis. *Front Pediatr*. 2017;5:191. http://doi.org/10.3389/fped.2017.00191

S7. Gertsman I, Johnson WS, Nishikawa C, et al. Diagnosis and monitoring of cystinosis using immunomagnetically purified granulocytes. *Clin Chem*. 2016;62(5):766-772. https://doi.org/10.1373/clinchem.2015.252494
